# Latent Autoimmune Diabetes in Adults: Background, Safety and Feasibility of an Ongoing Pilot Study With Intra-Lymphatic Injections of GAD-Alum and Oral Vitamin D

**DOI:** 10.3389/fendo.2022.926021

**Published:** 2022-07-18

**Authors:** Anneli Björklund, Ingrid K. Hals, Valdemar Grill, Johnny Ludvigsson

**Affiliations:** ^1^ Department of Molecular medicine and Surgery, Karolinska Institutet, Stockholm, Sweden; ^2^ Endocrine and Diabetes Unit, Karolinska University Hospital, Stockholm, Sweden; ^3^ Diabetes Center, Academic Specialist Center, Region Stockholm, Stockholm, Sweden; ^4^ Department of Endocrinology, Clinic of Medicine, St Olavs Hospital, Trondheim University Hospital, Trondheim, Norway; ^5^ Department of Clinical and Molecular Medicine, Norwegian University of Science and Technology (NTNU), Trondheim, Norway; ^6^ Nord-Trondelag Hospital Trust, Levanger, Norway; ^7^ Division of Pediatrics, Department of Biomedical and Clinical Sciences, Faculty of Medicine and Health Sciences, Linköping University, Linköping, Sweden; ^8^ Crown Princess Victoria Children´s Hospital, Linköping, Sweden

**Keywords:** latent autoimmune diabetes in adults, (LADA), antigen-specific immunotherapy, GADalum, intra-lymphatic, vitamin D

## Abstract

**Background:**

Latent Autoimmune Diabetes in Adults (LADA) constitutes around 10% of all diabetes. Many LADA patients gradually lose their insulin secretion and progress to insulin dependency. In a recent trial BALAD (Behandling Av LADa) early insulin treatment compared with sitagliptin failed to preserve insulin secretion, which deteriorated in individuals displaying high levels of antibodies to GAD (GADA). These findings prompted us to evaluate a treatment that directly affects autoimmunity. Intra-lymphatic GAD-alum treatment has shown encouraging results in Type 1 diabetes patients. We therefore tested the feasibility of such therapy in LADA-patients (the GADinLADA pilot study).

**Material and Methods:**

Fourteen GADA-positive (>190 RU/ml), insulin-independent patients 30-70 years old, with LADA diagnosed within < 36 months were included in an open-label feasibility trial. They received an intra-nodal injection of 4 μg GAD-alum at Day 1, 30 and 60 plus oral Vitamin D 2000 U/d from screening 30 days before (Day -30) for 4 months if the vitamin D serum levels were below 100 nmol/L (40 ng/ml). Primary objective is to evaluate safety and feasibility. Mixed Meal Tolerance Test and i.v. Glucagon Stimulation Test at baseline and after 5 and 12 months are used for estimation of beta cell function. Results will be compared with those of the recent BALAD study with comparable patient population. Immunological response is followed.

**Results:**

Preliminary results show feasibility and safety, with almost stable beta cell function and metabolic control during follow-up so far (5 months).

**Conclusions:**

Intra-lymphatic GAD-alum treatment is an option to preserve beta cell function in LADA-patients. An ongoing trial in 14 LADA-patients show feasibility and safety. Clinical and immunological responses will determine how to proceed with future trials.

## Introduction

LADA (Latent Autoimmune Diabetes in Adults) constitutes approximately 10% of all diabetes, yet best treatment is not known (1–3). LADA is usually defined as follows: onset of diabetes over 30 years of age, presence of autoantibodies against Glutamic acid decarboxylase (GADA), and/or other beta cell associated antibodies, and no need for insulin treatment during the first 3-6 months after diabetes has been diagnosed. The LADA population is quite heterogenous regarding phenotype with need for a flexible and personalized treatment (4).

Persons with LADA lose their ability to secrete insulin faster than those with type 2 diabetes but slower than type 1. In our opinion therapy should try to preserve insulin secretion as long as possible to facilitate treatment, improve blood glucose control and prevent both acute and late complications. When choosing treatment for persons with LADA it is necessary to consider in what stage of the disease the patient is (**Table 1**). Early, near diagnosis of the disease, life style advice and accompanying changes, and metformin is usually enough to maintain good metabolic control. Much later, usually years when insulin secretion is insufficient or absent, exogenous insulin is necessary. Best treatment for the period between early and late stages of the disease has been debated for many years. The hypothesis that beta cell rest by early insulin treatment can preserve beta cell function has been raised (1, 3). To test this hypothesis, we therefore performed an intervention trial, the BALAD trial, NCT01140438 (5). The aim of BALAD (Behandling Av (= treatment of) LADa) study was thus to investigate whether early insulin treatment at bedtime would preserve beta cell function better than per oral treatment with sitagliptin (5). Sitagliptin, a DPP-4 inhibitor exerts its anti-hyperglycaemic effects mainly through stimulation of insulin secretion, thus providing a treatment that contrasts with exogenous insulin, i.e., a beta-cell resting alternative. Providing such a contrast could, in theory at least, facilitate detection of a putative beneficial insulin effect. Another reason for choosing sitagliptin as comparator was its wide-spread use as add-on treatment for type 2 diabetes patients. Persons with LADA (n=64; 29 females and 35 males), who had been diagnosed less than 3 years ago were included. All were treated with metformin 2g/day or the highest tolerated dose. When insulin secretion started to decrease with signs of need for additional medication (middle phase in **Table 1**) they were randomized to either sitagliptin or bed-time insulin for 21 months as add-on-therapy to metformin. Inclusion criteria for sitagliptin or bedtime insulin treatment were set to approach a common situation in the clinic when single treatment with metformin was deemed not completely satisfactory: HbA1c > 10% of the upper limit of normal for HbA1c, positive GADA, fasting C-peptide > 0.3 nmol/L and no need for insulin for the last three months. Metabolic control (HbA1c) was similar in the two arms. In summary, the results showed that early insulin treatment could not preserve beta cell function better than sitagliptin Additionally – and importantly -, deteriorating insulin secretion with time was apparent, but seen preferentially in persons with high levels of autoimmunity, i.e. high levels of GADA

**Table 1:** Consensus regarding how to treat diabetes.

Diabetes type	From start	Later	Even later
Type 1 diabetes	Insulin	Insulin	Insulin
Type 2 diabetes	Life style, metformin	Anti-diabetic drugs	Anti-diabetic drugs incl. insulin
LADA	Life style, metformin	?	Insulin

Against the background of our results in the BALAD study it seemed clear that treatment more directly focused on the autoimmune process is needed to preserve insulin secretion in persons with LADA who displayed signs of high autoimmune activity. Which treatment modality should one then choose? It seemed natural that it should be based on current knowledge of intervention alternatives tested in type 1 diabetes with special consideration to the only mild symptoms that are typical at onset of LADA. A variety of immune interventions have been tried in Type 1 diabetes, but with limited success and sometimes significant and serious side effects (6–8). Anti-CD3 has been the most efficacious treatment (9, 10). However, Phase III trials failed (11–13). In subgroups of patients the treatment had some efficacy with best efficacy in patients under the age of 18 years (11, 12). Also, TNF-alfa inhibition (14, 15), ATG (Anti Thymocyte Globulin) (16), Alefacept (17) and Rituximab (18) had greater efficacy than placebo. However, these treatments have been burdensome for the patients with common, sometimes serious adverse events and risks, making such therapies less aattractive in LADA patients who, as mentioned above, usually have only mild symptoms at onset of their diabetes. However, long term complications are not less frequent in LADA than in other forms of diabetes (9), emphasising the need for good metabolic control throughout life. It can be surmised that simple, tolerable, safe treatments are as important in individuals with LADA as in type 1 diabetes. Autoantigen therapy then appears to be an interesting alternative (19, 20).

Thus, immunomodulation using autoantigen treatment to reduce or stop a destructive specific immune response seems to be less toxic and dangerous than heavier forms of immune suppression/modulation and can give a more specific response of the immune system than broader unspecific therapies (19). As GAD is the typical autoantigen towards patients with LADA, we found it natural to consider GAD-therapy as a possible approach.

The first study with GAD vaccination was a Swedish dose finding phase IIa study in 47 LADA patients. Here, the subcutaneous administration of various doses (4-500 µg) of GAD-alum were not associated with any adverse events. The study was not primarily designed to test for beneficial effects on insulin secretion (21, 22). No further studies on subcutaneous vaccination with GAD in a population of LADA patients have been published.

Trials in Type 1 diabetes using s.c. GAD-alum have given divergent results (23–25). However, a meta-analysis showed that this type of treatment was effective with >97% probability (26), even though the degree of efficacy was insufficient and needed to be improved. There are many unsolved questions in auto-antigen therapy (27) and one is the administration route. With regard to GAD-alum treatment intra lymphatic GAD-alum administration seems to be a safe, tolerable treatment which has given encouraging results both clinically and immunologically (28–31).

Against this background we therefore decided to test intra-lymphatic treatment with GAD-alum in persons with LADA who displayed strong signs of autoimmunity. In the current pilot study our primary objective was to evaluate safety and feasibility of this treatment regimen.

Secondary objectives were to test if the treatment induces a strong GAD-specific immune response similar to what has previously been observed in type 1 diabetes patients and to test for indications of preservation of endogenous insulin production.

## Material and Methods

### GADinLADA Study

Approval was obtained from the Medical Products Agencies and Ethical Board in Norway and the Swedish Ethical Review Authority.

The GADinLADA (NCT04262479) is an on-going open label phase IIa pilot trial aimed to include men and women aged 30-70 years. 14 participants were recruited from Trondheim (St Olavs Hospital, Trondheim University Hospital) and Stockholm (Center of Diabetes, Academic Specialist Center, Region Stockholm) with surroundings. Patients were given totally 3 intra-nodal injections of GAD-alum (one month apart) together with oral vitamin D.

The primary objective is to evaluate safety and feasibility of this treatment regimen. Secondary objectives are to test if the treatment induces a strong GAD-specific immune response similar to what has previously been observed in type 1 diabetes patients and to test for indications of preservation of endogenous insulin production. Here we present the study protocol and a few preliminary data 5 months after the last injection of GAD-alum.

#### Trial Design

##### Primary Endpoints

Variables for the Evaluation of Safety and Feasibility

1) Injection site reactions, skin reactions one hour post injection vs. before injection.
2) Occurrence of AEs, continuously registered and status summarized at 5 and 12 months after the first injection.
3) Laboratory measurements (haematology and clinical chemistry), status summarized at 5 and 12 months after the first injection vs. baseline.
4) Physical examinations, including neurological assessments, status summarized at 5 and 12 months after the first injection vs. baseline.
5) GAD65A titer in serum, levels at 5 and 12 months after the first injection vs. baseline.
6) Vital signs (blood pressure), status summarized at 5 and 12 months after the first injection vs. baseline.

##### Secondary Endpoints

Variables for the evaluation of beta cell insulin secretion capacity and metabolic control. Stimulation tests: glucagon-stimulated C-peptide test (GSCT) (32) and mixed meal tolerance test (MMTT) (33).

1) Insulin secretion measured by glucagon- and MMTT stimulated C-peptide at baseline, and at 5 and 12 months after the first injection.
2)- Change in HbA1c from baseline to 5 and 12 months after the first injection.
3) Change in fasting glucose from baseline to 5 and 12 months after the first injection.
4) Change in Fasting C-peptide between baseline and 5 and 12 months after the first injection.
5) Change in maximum C-peptide during MMTT between baseline and 5 and 12 months after the first injection.

Variables for the evaluation of immunological response at 5 and 12 months vs. baseline:

6) Measurement of serum concentration of GAD65-specific IgG1, IgG2, IgG3 and IgG4 antibodies for all included patients.
7) Measurement of supernatant concentrations of IL-1, IL-2, IL-5, IL-13, IL-10, IL-17, IFN-γ and TNF secreted during cultivation of PBMCs isolated from all included patients.
8) Characterization of PBMCs with FACS using CD3, CD4, CD8, CD45RA, CCR7, CD25, CD127, FOXp3 at baseline.
9) Analysis of the proliferation of PBMCs isolated from all included patients during cultivation with vehicle, GAD65 and control antibody.
10) Other relevant variables.

The schedule of the main study events is given in **Table 2**.

**Table 2:** Schedule of main events in the GADinLADA study.

Event	V1 Screening	V2		V3 Baseline		V4^a^ M1	V5 ^a^M2		V6 M5		V7 M12	
**DAY**	-44 to -60	-31	-30	1	2 (+2)	30 ( ± 5)	60 ( ± 5)	90	150 ( ± 14)	151 (+2)	360 ( ± 14)	361 (+2)
Informed consent	X	X										
Demographics	X											
GAD-alum (4 µg) ^a, b, c,^					X	X	X					
Vitamin D^d^ start/end			Start					Stop				
Neurological assessment	X			X		X	X		X		X	
Concomitant medication	X			X		X	X		X		X	
Vital signs (BP)	X			X		X	X		X		X	
Injection site inspection; investigator/study nurse^e^					X	X	X					
AEs				X		X	X		X		X	
Glucagon test^c,f^			X		X					X		X
MMTT^f^		X		X					X		X	
**Blood and urine sampling for safety, genetics, vitamin D levels and immunology:**												
*Hematology*	X			X		X	X		X		X	
*Clinical Chemistry*	X			X		X	X		X		X	
*GAD65A titer^g^*	X			X		X	X		X		X	
*HLA* *characterization*				X								
*Vitamin D level*	X	X		X		X	X		X		X	
*Other immunological parameters*		X		X		X	X		X		X	
*Creatinine*	X			X		X	X		X		X	
**Blood sampling for diabetes status:**												
*Fasting C-peptide*	X	X	X	X	X	X	X		X	X	X	X
*Fasting glucose*	X	X	X	X	X	X	X		X	X	X	X
*Glucagon stimulated Cpeptide and glucose*			X		X					X		X
*MMTT stimulated C-peptide*		X		X					X		X	
*MMTT stimulated glucose*		X		X					X		X	
*HbA1c*	X	X		X		X	X		X		X	

**Table 2**: V, visit; M, month; GAD, Glutamic Acid Decarboxylase; BP, Blood Pressure; AEs, Adverse Events; MMTT, Mixed Meal Tolerance Test; GAD65A, Glutamic Acid Decarboxylase

Antibodies; HLA, Human Leukocyte Antigen; HbA1c, Hemoglobin A1c].

^a^Study drug administration: For visit 4 and 5 the visit date must be set in accordance with visit 3 and 4, respectively, so that the first, second and third doses will be 30 days apart (± 5days).

^b^The GAD-alum injection directly into the inguinal lymph node is to be done by an appropriately qualified radiologist at the X-ray department at the study site by help of ultrasound technique.

^c^On day two at visit 2, the glucagon test needs to be done before the injection of GAD-alum.

^d^Supplementation with vitamin D starts at day -30, after the glucagon test has been performed, if the vitamin D serum levels are below 100 nmol/L (40 ng/ml) at screening. If the patient has vitamin D serum levels above 100 nmol/L (40 ng/ml) at screening, no Vitamin D supplementation will be given for that patient.

^e^The investigator/study nurse will inspect the injection site before and after the injection is given and record any injection site reactions in the Case Report Form (CRF).

^f^The MMTT and the Glucagon test must be carried out on separate days, as each test must be performed in the fasting state.

^g^Antibodies against IA-2 (islet cell antigen 2), ZnT8 (Zink transporter 8) and insulin will be measured at Baseline and at Visit 7.

#### Selection and *Withdrawal* of *Subjects*

##### Informed Consent

All study participants have received both written and oral information about the study procedures, potential risks, and benefits. Medications other than the study drug.

Antidiabetic medication in the form of metformin was accepted before and during the trial. Study participants had to be insulin independent at baseline, but if the need for insulin treatment developed during the trial, such treatment would be given. The need for insulin treatment was based on clinical judgement after considering the following parameters:

- Increased levels of HbA1c, i.e. two measurements, performed 1 month apart, showing an increase of 22 mmol/mol vs. baseline.
- Fasting blood glucose >10 mmol/L in at least 3 occasions during a single week.
- Weight loss >2 kg vs. baseline.

##### Inclusion Criteria

1. Signed informed consent by the patient.
2. Diagnosis of LADA within the last 18 months before inclusion. LADA was defined by the criteria of age ≥30 years at the onset of diabetes, anti-GAD positivity and no clinical need for insulin treatment during the first 3 months after the diagnosis of diabetes.
3. Male or female between 30-70 years of age
4. Fasting C-peptide levels ≥ 0.3 nmol/l
5. High GADA titers (>190 U/ml)
6. Patients had to be insulin independent at baseline by clinical judgement and C-peptide criteria
7. Antidiabetic medication in the form of metformin was acceptable for inclusion as well as medications not mentioned under exclusion criteria
8. Females agreed to avoid pregnancy and had a negative urine pregnancy test. Patients of childbearing potential must agree to use adequate contraception, until one (1) year after the last administration of GAD-alum. Adequate contraception is as follows:

For females of childbearing potential:

- oral [except low‐dose gestagen (lynestrenol and norestisteron)], injectable, or implanted hormonal contraceptives
- combined (estrogen and progestogen containing)
- oral, intravaginal or transdermal progesterone hormonal contraception associated with inhibition of ovulation
- intrauterine device
- intrauterine hormone-releasing system (for example, progestinreleasing coil)
- bilateral tubal occlusion
- vasectomized male (with appropriate post vasectomy documentation of the absence of sperm in the ejaculate)
- male partner using condom
- abstinence from heterosexual intercourse

For males of childbearing potential:

- condom (male)
- abstinence from heterosexual intercourse

##### Exclusion Criteria

1. Current or previous treatment with immunosuppressant therapy (topical or inhaled steroids are accepted).
2. Continuous treatment with anti-inflammatory drug (sporadic treatment e.g. because of headache or in connection with fever a few days will be accepted).
3. Systemic treatment with glucocorticoids.
4. Treatment with any vaccine, including influenza vaccine, within 1 month prior to planned first study drug dose or planned treatment with any vaccine up to 1 month after the last injection with study drug.
5. Antidiabetic medication (metformin excepted).
6. Significantly abnormal hematology results at screening (i.e. anemia with hemoglobin < 12 g/L).
7. A history of epilepsy, head trauma or cerebrovascular accident, or clinical features of continuous motor unit activity in proximal muscles.
8. Clinically significant history of acute reaction to vaccines in the past.
9. Renal disease (as defined by serum creatinine >150 µmol/l).
10. Serious cardiovascular events (myocardial infarction, stroke) within the last year preceding recruitment.
11. Participation in other clinical trials with a new chemical entity within the previous 3 months.
12. A history of alcohol or drug abuse.
13. Known HIV or hepatitis.
14. Presence of associated serious disease or condition, including active skin infections that preclude intralymphatic injection, which in the opinion of the investigator makes the patient non-eligible for the study.
15. Other serious chronic disease as judged by investigator.
16. Females who are lactating, are pregnant or intend to become pregnant.
17. Inability or unwillingness to comply with the provisions of this protocol.
18. Deemed by the investigator not being able to follow instructions and/or follow the study protocol.
19. Treatment with any other supplementation of vitamin D, marketed or not, or unwilling to abstain from such medication during the 120 days daily intake of Divisun (non-investigational medicinal product).

##### Criteria for Terminating the Investigational Product Treatment/Trial Treatment

Rapid increase in HbA1c or decline in C-peptide levels vs. historical controls. Evidence for induction of stiff person syndrome (SPS) or any adverse neurological or behavioural effects.

#### GAD-Alum Should Not Be Given to the Patient if the Patient After Inclusion in the Study Develops/Experiences

- Brain damage, epilepsy, head trauma, neurological disease
- Any active, serious hormonal disease other than LADA
- Other severe autoimmune disease (except celiac disease which is accepted for inclusion)
- Immune-suppressive treatment
- Cancer, cancer treatment
- Any vaccination
- Drug/alcohol abuse
- Becomes pregnant or is no longer willing to use safe contraceptives during the study

##### Vitamin D Supplementation Should Not Be Continued if the Patient After Inclusion in the Study Develops/Experiences:

- Symptoms of hypercalcemia such as tiredness, euphoria, drowsiness, nausea, weight loss, thirst, polyuria, nefrocalcinosis, renal failure
- Arrhythmia
- Pancreatitis

#### Study Treatment and Procedures

Patients were given three intra-lymphatic injections with 4 µg GAD-alum on days 2, 30 and 60. They also received 2000 IE oral vitamin D for 4 months (day-30 -90) if serum vitamin D was <100 nmol/L at screening (D-30) (for detailed study design see **Table 2**). Injections were given by a qualified radiologist at the X-ray department of the study site department by help of the ultrasound technique. The responsible investigator or study nurse inspected the injection site prior to administration of GAD-alum. The patient remained in the vicinity of the study site for the next hour after the injection, and the injection site was investigated again by the responsible investigator or study nurse one hour post injection. Attempts were made to inject GAD-alum into the same lymph node each time. The time of the day for injection will be decided by the cooperating radiologist.

#### Assessment of Efficacy

Follow-up visits will be performed after 150 and 360 days. Medical examination isperformed at all visits including baseline (day1) and months 1, 2, 5 and 12.

Endogenous Insulin Production will be measured by glucagon- and mixed meal stimulation of C-peptide at baseline and after 5 and 12 months of intervention. Metabolic control will be evaluated by measurements of HbA1c and fasting glucose at all study visits. Immunological response will be assessed by measurements of GADA and the immunological parameters listed in secondary endpoints 6-10 above. Blood samples for the assessment of immunological response will be collected at all study visits. Mixed meal tolerance test (MMTT (34) will be performed one month before baseline (day-30), at baseline (day 1) and at months 5 and 12.

The MMTT (33) and the GSCT (32) must be carried out on separate days, as each test must be performed in the fasting state. Also, if the patient develops need for insulin treatment during the study period, the patient must not take short acting/direct acting insulin within 6 hours before the test. The patient is allowed to take basal-insulin the day/night before, but not in the test morning and during the MMTT and the GSCT. If the patient does not fulfill the abovementioned criteria, the MMTT and GSCT should be rescheduled and the patient return to the study site within 5 days if possible.

##### Procedures for the MMTT and GSCT : MMTT

The patient will be given a standardized liquid nutrition mixture. Blood samples will be secured for glucose and C-peptide measurements at before and 30, 60, 90 and 120 ( ± 5) minutes after the meal. Mean C-peptide is assessed as the Area under the curve (AUC) in the first 120 minutes of the MMTT, calculated by the trapezoid rule in units of nmol/L/120min.

##### GSCT

The patient will receive an intravenous injection of 0.5 mg glucagon. Blood samples will be secured both before and 6 minutes after the injection for the measurements of glucagon-stimulated C-peptide and glucose.

Glucagon stimulated C-peptide tests (35) will be performed at the same time points as the MMTT (two consecutive days). Injection site is inspected after every injection and adverse events continuously recorded throughout the study.

##### HLA Genotyping

HLA genotyping was performed at baseline (day 1) and analysed at Oslo University hospital Rikshospitalet Oslo, Norway. Haematology, clinical chemistry, urine and HbA1c have been and will be analysed at local laboratories (St. Olavs hospital, Trondheim University Hospital and Karolinska lab in Stockholm). GADA, IA-2A, ZnT8A, IAA, and C-peptide will be measured at one common laboratory.

##### Immunological Studies

Immunological studies are performed at Linköping university, Sweden. For cytokine quantification, peripheral blood mononuclear cells (PBMC) will be cultured for 7 days in the presence of 5 µg/mL recombinant human GAD65 (Diamyd medical) or in medium (AIM-V) alone at 37C in 5% carbon dioxide, as previously described (36). Interleukin-10 (IL-10) and IL-13 will be measured in cell culture supernatants using the Bio-Plex Pro Cytokine panel (Bio-Rad, Hercules, CA) according to the manufacturer’s instructions. Data will be collected using the Luminex 200. The-antigen-induced cytokine secretion level will be calculated by subtracting the spontaneous secretion (i.e., secretion from PBMC culture in medium alone) from that following stimulation with GA65.

To quantify PBMC proliferation, PBMC will be resuspended in AIM-V medium and incubated in triplicate in round-bottomed 96-well plates with 5 µg/mL recombinant human GAD65 (Diamyd medical) or in AIM-V medium alone. After 3 days, cells will be pulsed for 18 h with 0.2 µCi of [3H] thymidine per well (Perkin Elmer) and thereafter harvested. Proliferation will be recorded using a cell counter and expressed as stimulation index, calculated as the median of triplicates in presence of stimulus divided by the median of triplicates with medium alone.

#### Safety and Adverse Events

The safety assessments include occurrence of adverse events (AEs), laboratory measurements, physical examinations including neurological assessments. Adverse events will be recorded by the physician at every visit throughout the study.

##### Blood Tests for Safety

- Chemistry: Creatinine, Calcium, Liver function tests (alanine aminotransferase (ALT), aspartate aminotransferase (AST), alkaline phosphatase, bilirubin)
- Haematology (MHC, MCV, MCHC, Haemoglobin, Platelets, Leukocytes)

##### Urine Analysis for Safety

- Urine pregnancy test as appropriate
- Microalbuminuria
- Creatinine

###### Other Variables Which Will Be Evaluated

- Inflammatory markers, especially TNF-alfa, IL-1 beta, IL-2, IL-17
- Th2-deviation of cell-mediated immune response seen e.g. as increased ratio of IL-5,10, 13 in comparison with IFN-gamma, TNF-alfa, IL-1 beta and IL-17, and increase of T-regulatory cells
- C-peptide (90-minute value and AUC mean 0-120 min) during a MMTT
- Fasting C-peptide
- HbA1c
- Vitamin D

### Definitions of Adverse Events and Reactions

An adverse event (AE) is defined as any untoward medical occurrence in the clinical trial subject administered a medicinal product and which does not necessarily have a causal relationship with this treatment. An adverse event can therefore be any unfavorable and unintended sign (including an abnormal laboratory finding, for example), symptom or disease temporally associated with the use of a medicinal product, whether or not considered related to the medicinal product.

A serious adverse event (SAE) is defined as any untoward medical occurrence or effect that at any dose results in death, is life-threatening, requires hospitalization or prolongation of existing hospitalization, results in persistent or significant disability or incapacity, or is a congenital anomaly or birth defect.

These characteristics/consequences have to be considered at the time of the event. For example, regarding a life-threatening event, this refers to an event in which the subject was at risk of death at the time of the event; it does not refer to an event which hypothetically might have caused death if it were more severe.

Some medical events may jeopardize the subject or may require an intervention to prevent one of the above characteristics/consequences. Such events (referred to as *important medical events*) should also be considered as serious in accordance with the definition.

A suspected unexpected serious adverse reaction (SUSAR) is defined as a SAE, the nature or severity of which is not consistent with the applicable product information (e.g.

investigator’s brochure for the unauthorized investigational product).

The term ‘severity’ is used here to describe the intensity of a specific event. This has to be distinguished from the term ‘serious’. The term severe is used to describe the intensity (mild, moderate or severe) of the event and the event does not necessarily need to be considered serious. The term serious is based on the patient/event outcome or action and serves as a guide for defining regulatory reporting obligations.

Mild intensity: The adverse event is transient and easily tolerated.

Moderate intensity: The adverse event causes the patient discomfort and interrupts the patient’s usual activities.

Severe intensity: The adverse event causes considerable interference with the patient’s usual activities and may be incapacitating or life-threatening.

Relationship to study medication

Relationship to study medication will be assessed for the two treatments (Diamyd and Vitamin D) separately. AEs with a causal relationship assessment of Unlikely related, Possibly related and Probably related will be considered to be Adverse Reactions.

Not related: This category is applicable to those AEs which, after careful medical consideration at the time they are evaluated, are judged to be clearly and incontrovertibly due to extraneous causes (disease, environment, etc.) and do not meet the criteria for study medication relationship listed under remote, plausible or probable.

Unlikely related: Time relationship non-existent or doubtful and/or other factor(s) certain or probable to have been causative.

Possibly related: Time relationship exists. Other possible causative factor(s) may exist (e.g., concurrent disease or concomitant medication). Improvement on dechallenges or dose reduction may or may not have been seen.

Probably related: Time relationship exists. No other possible causative factor(s) may exist (not reasonably explained by the patient’s known clinical state or concomitant medication). Improvement on dechallenges or dose reduction (if performed) has occurred. Recurrence of symptoms on rechallenge (if performed) has occurred. A specific laboratory investigation (if performed) has confirmed the relationship.

Reporting and follow up of adverse and serious adverse events (AE and SAE):

All AE must be recorded in the CRF, defining relationship to study medication, severity and seriousness. AE should also be recorded by the physician or study nurse in the patient file/notes.

#### Timelines and Reporting of SAE

All SAEs must be reported, whether or not considered attributable to the study drug on a separate SAE Report Form. SAEs will be reported from signing of informed consent.

TFS Trial Form Support AB (TFS) will be responsible for reporting all SAEs in accordance with ICH Good Clinical Practice (GCP) and local regulations. Diamyd and TFS will complete and sign a “Pharmacovigilance Working Agreement” agreement covering the safety reporting responsibilities in the study. This agreement will ensure the sponsor is directly informed of each SAE reported by the investigators.

It is the investigator’s responsibility to, as soon as he/she is aware of a potential SAE, contact TFS by fax or e-mail, and in any case no later than 24 hours after the knowledge of such a case.

Fatal and life-threatening SUSARs should be reported by TFS as soon as possible to the Competent Authorities and Ethical Committees in Norway and Sweden, and in any case no later than seven (7) calendar days, after knowledge by the Sponsor/TFS of such a case. Relevant follow-up information on the case will be subsequently communicated within an additional eight (8) days. All other SUSARs shall be reported to the Competent Authorities concerned and to the Ethics Committee concerned as soon as possible but within a maximum of fifteen (15) days of first knowledge by TFS.

### Statistics

This is a pilot study with no placebo arm. All research persons have received active substance i.e. intra-lymphatic GAD-alum. Study results will be compared to historical controls from the BALAD study which had a similar population of study participants.

#### Statistical Analysis Plan

In brief the following analyses are planned:

All continuous variables will have the following descriptive statistics displayed: Number of observations (n), mean value, standard deviation, minimum, median and maximum. All variables of a categorical nature will be displayed with frequencies and percentages. The tabulation of the descriptive statistics will be split by visit. Where appropriate, baseline (screening) descriptive statistics will also be included.

Demographic and other baseline characteristics will be presented using descriptive statistics (summary tables).

##### Variables

The AE/SAE data will be presented using a standardized tabulation of the frequency and incidence rate of all observed AE/SAEs. The frequencies and incidence rates are calculated on a per patient basis. Adverse events will be summarized by body system, causality and severity. Other safety data will be presented as descriptive statistics.

Data regarding immune response, beta cell function, AE and other data will be summarized descriptively.

Analyses of data will be performed after 5 and 12 months from baseline. Data from the BALAD study (see above) will be used as a historical control for the evaluation of treatment related effects on the evolution of beta cell function parameters between baseline and 5 and 12 months of intervention. We acknowledge that it is difficult to statistically compare data from BALAD as a historical control with data from this pilot study, as the participants in BALAD were randomized to active treatment. The comparison will still be valuable, primarily as a pointer for future research.

#### Preliminary Interim Results

**Figure 1:**
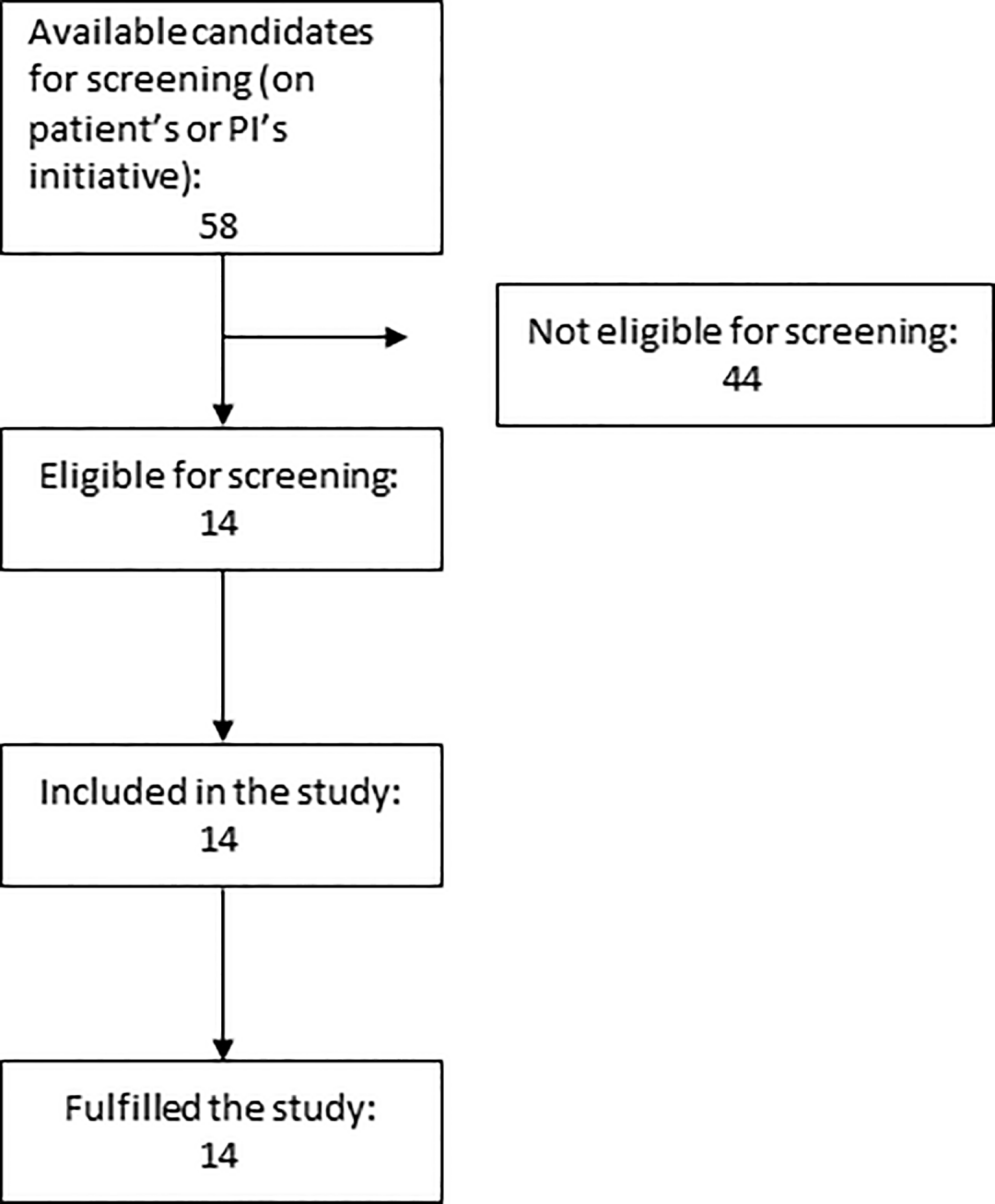
Flow Chart showing the number of patients approached, eligible and later included in the trial.

In total 14 participants (30-70 years) with LADA diagnosed less than 18 months before the study were recruited, eight in Stockholm and six in Trondheim. Study flow chart is shown in **Figure 1**. and basal characteristics of participants in **Table 3**. Mean BMI was in the overweight category and metabolic control was excellent, HbA1c = 42 ± 7 mmol/mol.

**Table 3:** Characteristics of the study population.

Study population characteristics at the time of inclusion	
Study participants	14
Study participants, Norway/Sweden	6/8
Male/female	7/7
Age at inclusion; years Mean (SD)	47 (8)
Age at inclusion; years range	30 - 62
Time from diagnosis to inclusion; months Mean (SD)	5.9 (3.8)
BMI; kg/m^2^ Mean (SD)	26.5 (5.2)
BMI; kg/m^2^ range	19.2 – 36.7
Metformin before inclusion	9/14
Fasting C-peptide; nmol/L Mean (SD)	0.65 (0.36)
Syst. BP	119
Diast. BP	76
HbA1c; mmol/mol Mean (SD)	43 (7)
Anti-GAD titer; units/ml Mean (SD)	157633 (550593)
HLA-DR3DQ2 positive/negative	7/7

SD, standard deviation; BMI, Body Mass Index; HLA, Human Leucocyte Antigen.

**Figure 2:**
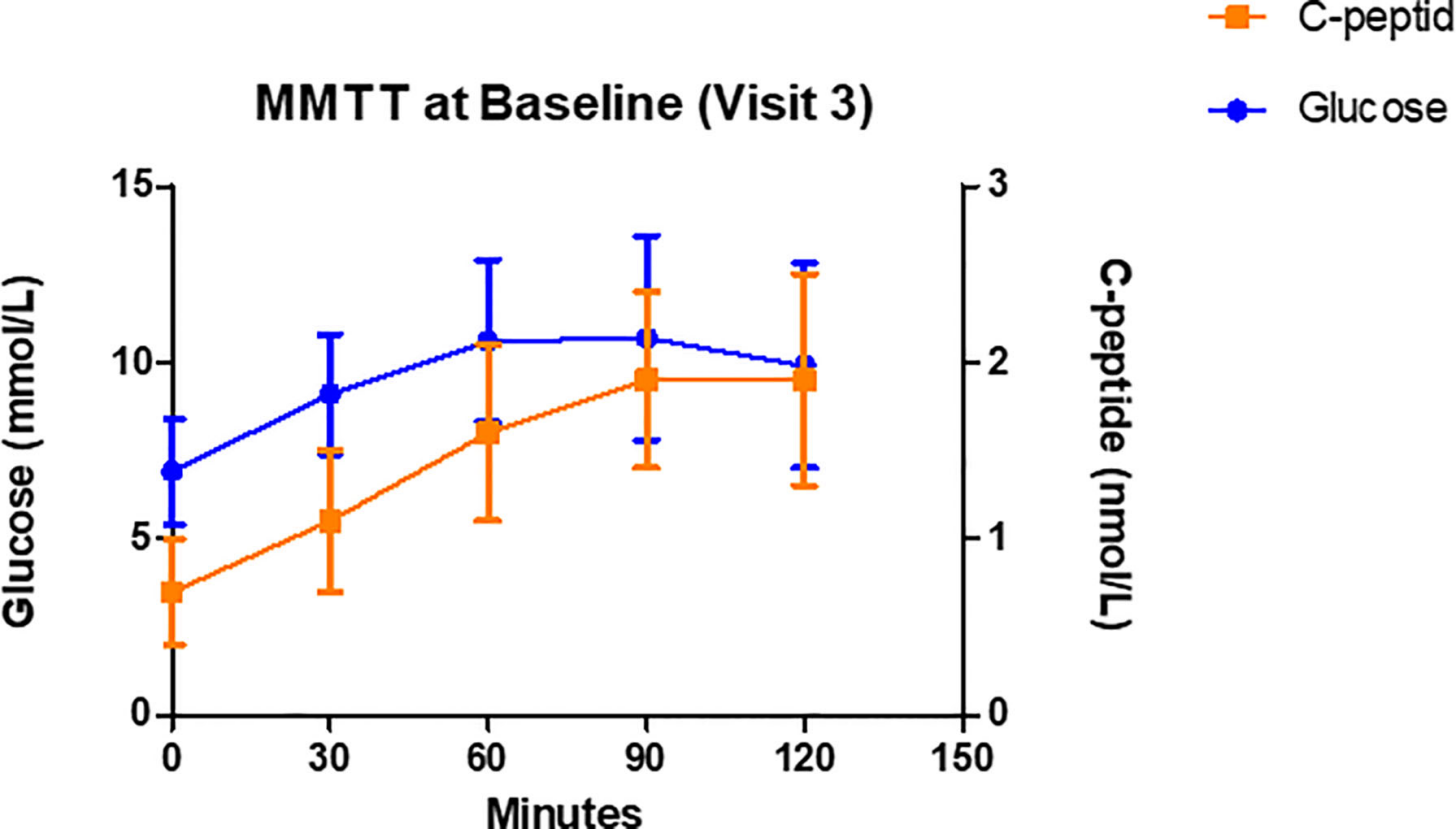
C-peptide and glucose levels during Mixed Meal Tolerance Tests (MMTT) performed at Baseline. Data are mean ± SD, n=14.

According to previous studies (31, 37) patients carrying the HLA DR3-DQ2 seem to be those who respond best to GAD-alum treatment. These studies were however not available at the time GADinLADA was started. The rationale for including analyses of HLA in participants in the GADinLADA study was the known shared frequency between LADA and type 1 diabetes of HLA genes conferring either susceptibility or protection to type 1 diabetes. As shown in **Table 3** seven out of totally fourteen patients carry the HLA DR3-DQ2 haplotype. **Figure 2** shows glucose and C-peptide values measured after MMTT at baseline. In **Table 4** mean insulin secretion after GSCT is shown at baseline and after 5 months.

**Table 4:** Insulin secretion capacity at Baseline and Month 5 assessed by GSCT

Glucagon-Stimulated C-peptide Test (GSCT)				
		Fasting	6 min>	Increment
		C-peptide (nmol/L)		
Baseline	n	14	14	14
	Mean (SD)	0.73 (0.44)	1.14 (0.53)	0.41 (0.37)
	Median	0.65	1.20	0.35
	Q1, Q3	0.38, 0.98	0.68, 1.55	0.20, 0.63
Month 5	n	14	14	14
	Mean (SD)	0.68 (0.40)	1.05 (0.42)	0.37 (0.21)
	Median	0.50	1.00	0.35
	Q1, Q3	0.40, 0.95	0.68, 1.40	0.20, 0.50
P-value for comparison^1^ to Baseline		0.296	0.395	0.582

^1^Wilcoxon signed rank test. Data are mean ± SD, n=14.

Preliminary results after 5 months show feasibility and no safety concerns. Thus, there have been no treatment-related serious adverse events and only minor transient reactions at the injection sites. So far, no patient has needed to start insulin treatment.

## Discussion

Persons with LADA who display signs of high autoimmunity lose their residual insulin secretion more rapidly than those with phenotypic Type 2 diabetes, and consequently become earlier insulin-dependent (32, 33). Epidemiological studies indicate that metabolic control in an early period after the onset of LADA diabetes is acceptable but that metabolic control deteriorates with time and is accompanied by long-term complications (38) Such findings seem to highlight the importance of residual beta cell function. To find treatment to preserve residual beta cell function is therefore of crucial importance. Such treatment needs to be safe and tolerable for the patients as LADA patients have reasonably good prognosis and quality of life already with conventional treatment. As LADA patients usually have autoantibodies against GAD, and GAD has been used in autoantigen treatment with extremely good safety profile and some efficacy, it has been natural to try such treatment.

The DIAGNODE- 1 trial showed promising efficacy (28) and in the DIAGNODE 2 study, with similar design,which we also use in the GAD-in LADA study, persons with recent onset type 1 diabetes and HLA haplotype DR3-DQ2 showed preservation of beta cell function after intra-lymphatic injections of GAD-alum (31). No serious side effects were shown in the previous DIAGNODE studies. Half of LADA patients are highly autoimmune with high GADA levels, and approx. 50% of them also have other autoantibodies (5). Preliminary data indicates that half of the highly autoimmune LADA patients carry the HLA DR3-DQ2 genotype (Elin Sørgjerd, personal communication) which according to previous studies seem to be those who respond best to GAD-alum treatment (31, 37). This motivates similar treatment in a larger clinical trial in LADA patients who display signs of high autoimmunity.

In conclusion, therapies to preserve beta cell function are warranted in LADA patients. Due to the “mildness” of the LADA phenotype the treatment has to be rather simple and very safe. Intra-lymphatic GAD-alum treatment parallel with oral Vitamin D substitution seems to be a feasible, safe and tolerable approach, and might be a way to improve the course of the disease.

## Data Availability Statement

The original contributions presented in the study are included in the article. Further inquiries can be directed to the corresponding author.

## Ethics Statement

The studies involving human participants were reviewed and approved by the Regional Ethical Committee, Central Norway and the Swedish Ethical Review Authority. The patients/participants provided their written informed consent to participate in this study.

## Author Contributions

AB initiated the study in Sweden, recruited and followed patients and wrote the manuscript. IKH supervised the study, recruited and followed participants, analysed results and revised the manuscript. VG designed and initiated the study in Norway and took part in writing the manuscript. JL was involved in the design, critically reviewed and took part in writing the manuscript. All authors approved the final version of the manuscript. All authors contributed to the article and approved the submitted version.

## Funding

The study is funded by Central Norway Regional Health Authority (Stjørdal, Norway), St Olavs hospital (Trondheim University Hospital, Trondheim, Norway) and the Norwegian Diabetes Association.

## Conflict of Interest

The authors declare that the research was conducted in the absence of any commercial or financial relationships that could be construed as a potential conflict of interest.

## Publisher’s Note

All claims expressed in this article are solely those of the authors and do not necessarily represent those of their affiliated organizations, or those of the publisher, the editors and the reviewers. Any product that may be evaluated in this article, or claim that may be made by its manufacturer, is not guaranteed or endorsed by the publisher.

## Abbreviations

AE, Adverse Event; ALT, Alanine aminotransferase; AST, Aspartate aminotransferase; AUC, Area Under the Curve; BALAD, Behandling Av LADA (Treatment of LADA); CCR, CC Chemokine Receptor; CD, Cluster of Differentiation Antigen; CRF, Case Report Form; CTLA-4, Cytotoxic T-Lymphocyte Associated Protein 4; DPP-4, Dipeptidylpeptidase-4; eCRF, Electronic Case Report Form; FOXp3, Forkhead box p3; GAD, Glutamic Acid Decarboxylase; GADA, Glutamic Acid Decarboxylase Antibody; GCP, Good Clinical Practice; GSCT, Glucagon-Stimulated C-peptide Test; HbA1c, Hemoglobin A1c; HIV, Human Immunodeficiency Virus; HLA, Human Leucocyte Antigen; ICH, International Council for Harmonisation; Ig, Immunoglobulin; IL, Interleukin; IFN, Interferon; LADA, Latent Autoimmune Diabetes in Adults; MCHC, Mean Cell Hemoglobin Concentration; MCV, Mean Corpuscular Volume; MHC, Major Histocompatibility Complex; MMTT, Mixed Meal Tolerance Test; NPH, Neutral Protamine Hagedorn; PBMC, Peripheral Blood Mononuclear Cells; RHA, Regional Health Authority; SAE, Serious Adverse Event; SDS, Source Data Verification; SPS, Stiff Person Syndrome; SUSAR, Suspected Unexpected Serious Adverse Reaction; TFS, Trial Form Support AB; Th2, T helper type 2; TNF, Tumor Necrosis Factor.
